# Making a virtue of necessity: the pleiotropic role of human endogenous retroviruses in cancer

**DOI:** 10.1098/rstb.2016.0277

**Published:** 2017-09-11

**Authors:** George Kassiotis, Jonathan P. Stoye

**Affiliations:** 1 Retroviral Immunology, The Francis Crick Institute, London, UK; 2 Retrovirus–Host Interactions, The Francis Crick Institute, London, UK; 3 Department of Medicine, Faculty of Medicine, Imperial College London, London, UK

**Keywords:** endogenous retroviruses, cancer, immunogenicity

## Abstract

Like all other mammals, humans harbour an astonishing number of endogenous retroviruses (ERVs), as well as other retroelements, embedded in their genome. These remnants of ancestral germline infection with distinct exogenous retroviruses display various degrees of open reading frame integrity and replication capability. Modern day exogenous retroviruses, as well as the infectious predecessors of ERVs, are demonstrably oncogenic. Further, replication-competent ERVs continue to cause cancers in many other species of mammal. Moreover, human cancers are characterized by transcriptional activation of human endogenous retroviruses (HERVs). These observations conspire to incriminate HERVs as causative agents of human cancer. However, exhaustive investigation of cancer genomes suggests that HERVs have entirely lost the ability for re-infection and thus the potential for insertional mutagenic activity. Although there may be non-insertional mechanisms by which HERVs contribute to cancer development, recent evidence also uncovers potent anti-tumour activities exerted by HERV replication intermediates or protein products. On balance, it appears that HERVs, despite their oncogenic past, now represent potential targets for immune-mediated anti-tumour mechanisms.

This article is part of the themed issue ‘Human oncogenic viruses’.

## 1. Introduction

Our increasing understanding of the aetiology of cancer has implicated viral infection as the direct cause of as many as one in five human cancers [1]. Notable examples include Epstein–Barr virus, hepatitis B and C viruses, human immunodeficiency virus type 1 (HIV-1) together with the human herpes virus 8 (HHV-8), human papilloma virus, Merkel cell polyomavirus and human T-cell leukaemia virus type 1 (HTLV-1), which are reviewed extensively in this special volume.

The concept of a cancer-causing virus dates back to the landmark discovery of Rous sarcoma virus (RSV), an infectious oncogenic retrovirus inducing sarcomas in domestic fowl [2]. It was indeed the study of transmissible animal retroviruses such as RSV and avian leukosis virus in domestic fowl, as well as murine leukaemia virus and mouse mammary tumour virus in laboratory mice that established the first principles in cancer research [3] and forged a strong link between retroviruses and cancer in the mindset of the research community [4].

Notably, the study of the same animal retroviruses and the cancers they were causing in animals eventually led to the discovery of endogenous retroviruses (ERVs) [5–7], reinforcing the link between retroviruses and cancer. This putative link developed almost into an obsession in the research community, who began hunting for retroviruses, exogenous as well as endogenous, as causative agents of many different types of cancer and other human conditions [8]. This effort was further fuelled by the awareness of the huge number of human endogenous retroviruses (HERVs) in our germline that the Human Genome Project had revealed [9], and their frequent transcriptional activation in cancer [10–13].

Human cancer can undoubtedly result from retroviral infection. This is exemplified by infection with exogenous retroviruses, such as HTLV-1 which causes adult T-cell leukaemia/lymphoma and HIV-1 which predisposes to Kaposi sarcoma. The development of leukaemias following gene therapy with early versions of retroviral vectors also highlighted the oncogenic potential of these vectors in humans [14]. Also certain is the ability of ERVs to cause cancer in a variety of animal species other than humans. Indeed, ERV activity may largely explain Peto's paradox, that the incidence of cancer, at the species level, is fixed and does not appear to correlate with the number of cells in an organism [15].

However tempting, the hypothesis that HERVs also cause human cancer is, nevertheless, still based on relatively limited experimental evidence and should only be taken as a starting point for further investigation. Interestingly, this investigation has also uncovered a less appreciated facet of HERV biology as contributors to host defence against tumours. Here, we provide an assessment of the available data from studies looking into the potential involvement of HERVs in cancer development. Based on the accumulating evidence, we would suggest that these once infectious and potentially oncogenic agents have now been conscripted to help protect the host.

## 2. What distinguishes human endogenous retroviruses from other retroelements?

Complete or partial HERV provirus fragments in the human genome are recognized based on sequence homology with exogenous retroviruses. They share the canonical approximately 9000 base pair (bp) retroviral genomic structure, where the *gag*, *pol* and *env* genes are flanked by two long terminal repeats (LTRs), a structure that gives away their evolutionary origin. Indeed, HERVs originate from distinct events of infection of the human germline by exogenous retroviruses, followed by waves of further copy number amplification, by either germline re-infection or retrotransposition [16–18]. The multitude of germline invasion events has created considerable diversity in HERVs, which are generally grouped into three distinct classes (Class I, II and III), according to sequence similarity with different groups of exogenous retroviruses [19]. Further subgroups of HERVs are recognized, conventionally depending on and named after the amino acid that corresponds to the tRNA primer predicted to initiate reverse transcription of the respective HERV RNA genome, or by sequence similarity of particular open reading frames [20,21], although a revised nomenclature for HERVs has been proposed [22].

Over long evolutionary time periods, most HERVs have suffered substantial mutational damage, with only a few copies retaining some of the open reading frames [16,18,21,23]. By contrast, more recently acquired HERV proviruses may have intact open reading frames for all their genes [24]. These are likely to have avoided fixation in the human germline and are thus insertionally polymorphic [25,26], although relatives of insertionally polymorphic HERVs are also found in other species [27]. Collectively, HERVs make up to 4.77% of the human genome [9], but they are not the only group of LTR elements in our genome. Also, bound by LTRs, the mammalian apparent LTR retrotransposons (MaLRs) make up an additional 3.78% of the human genome [9] and represent smaller elements (typically approx. 3000 bp in length) that have lost replication autonomy.

An even larger part of the human genome is occupied by retrotransposons that are not bound by LTRs [28–30]. These non-LTR elements are distinguished into long and short interspersed nuclear elements (LINEs and SINEs), comprising 20.99% and 13.64% of the human genome, respectively [9]. As the name suggests, LINEs are longer retrotransposons (approx. 6000 bp for the canonical LINE1 element) and have retained the ability for autonomous retrotransposition [30]. By comparison, SINEs are much smaller in size and cannot retrotranspose autonomously [31]. Instead, retrotransposition of SINEs is completed by use of the LINE1-encoded reverse-transcriptase (ORF2p) [31]. LINEs are responsible for the unsurpassed retrotransposition frequency of *Alu* elements, a primate-specific type of SINE [31]. Restricted to hominids and also dependent on LINEs for retrotransposition are the composite SVA elements (SINE-VNTR-Alu), which are particularly active [32,33].

Although the primary focus of this review is on the potential contribution of LTR elements (HERVs and MaLRs) to cancer development, a comparison with non-LTR retrotransposons will also be made, to illustrate the scale of the risk they each pose.

## 3. The case for the prosecution: oncogenic properties of human endogenous retroviruses

Studies with infectious retroviruses have pinpointed insertional mutagenesis as the principal mode of oncogenesis [4]. However, retroviruses may affect genome structure or function by additional mechanisms, which are examined separately.

### (a) Insertional mutagenesis

Insertional activation of oncogenes or disruption of tumour-suppressor genes during retroviral infection requires the completion of the retroviral life cycle, with insertion of the proviral DNA into the host cell DNA. This process creates a new copy of the HERV provirus, which our current sequencing tools should be able to detect. To date, no such new HERV integration has been found, suggesting that HERVs are no longer replication-competent. This may in part be due to the accumulated degeneracy of open reading frames in the majority of HERVs.

Between them, non-polymorphic HERV-K(HML-2) proviruses carry functional open reading frames for all the necessary components to create a transducing particle, although replication of a reconstructed HERV-K(HML-2) virus was limited [34,35]. Recombination between defective proviruses can bring together functional open reading frames, ultimately restoring infectivity, as has been demonstrated in mouse cancer cell lines [36,37] or in immunodeficient mouse colonies *in vivo* [38,39]. Analysis of the human germline indicates that HERV-K(HML-2) proviruses can also recombine *in vivo* [25,40]. Moreover, somatic recombination between HERV-K(HML-2) proviruses may be facilitated by infection with HIV-1, likely through transactivation of proviral transcription by HIV accessory proteins or HERV-K(HML-2) genome mobilization [41]. Nevertheless, the spontaneous generation of a replication-competent HERV-K(HML-2) retrovirus through recombination between defective precursors has not yet been observed. It is possible that, in addition to open reading frame degeneracy or other less obvious changes, replication of HERV-K(HML-2) viruses in human cells may be inhibited by a cellular factor that restricts a post-entry, pre-integration step [42].

In addition to fixed proviruses, insertionally polymorphic HERV-K(HML-2) proviruses also carry intact open reading frames in some or all of their genes, often with fewer mutations than the older fixed counterparts [25,26,33,43]. The discovery of new insertionally polymorphic HERV-K(HML-2) proviruses, not previously annotated, has recently been reported [26,44,45]. These include an X-linked HERV-K(HML-2) provirus, present at very low frequency in the human population, with no apparent sequence mutations that might affect infectivity [26]. This discovery raises the possibility that rare polymorphic HERV-K(HML-2) proviruses are replication-competent [26]. However, no *de novo* integrations of any such retrovirus have yet been detected in the same datasets. Moreover, sequencing of approximately 2500 whole cancer genomes in the past 10 years has not identified any somatic HERV integrations that could have resulted from HERV re-infection of cancer cells [44,46].

The failure to find novel proviruses might simply reflect an inability to detect new integrations. However, this seems unlikely because somatic retrotransposition of non-LTR retrotransposons in humans is amply demonstrated [28,33,47]. Several hundreds of somatic LINE1 retrotranspositions have been demonstrated in a variety of human cancers, including colorectal, prostate and ovarian [44], Barrett's oesophagus and oesophageal carcinoma [48], gastric and pancreatic [49,50], but perhaps interestingly not in blood or brain cancers [44]. Moreover, LINEs can also mobilize non-repetitive downstream DNA sequences, a process known as 3′ transduction that is also potentially mutagenic [51]. In addition to LINE1 retrotranspositions, which were found in about half of cancer samples, 3′ transductions were also found in approximately a quarter of the same samples [52]. Lastly, a small proportion of human cancer samples (approx. 6%) were also found to carry somatically acquired pseudogenes, products of somatic retrotransposition of host messenger RNAs by the LINE1 machinery [53,54]. Thus, although not always definitively proven oncogenic, non-LTR element retrotransposition, as well as mobilization of non-repetitive DNA sequences by the LINE1 machinery, is a very frequent event in human cancer.

### (b) Chromosomal rearrangement

Another common feature of cancer genomic alterations is chromosomal rearrangements, which often play a causative role. Deletions, duplications and translocations can result from non-allelic homologous recombination [55] and copies of near identical HERV proviruses or even solitary LTRs scattered throughout the genome could, in principle, direct genomic recombination between such non-allelic loci [17,40,56]. The study of HERV-K(HML-2) proviruses has provided evidence for germline non-allelic recombination events [40]. Indeed, *de novo* non-allelic recombination between two highly similar (94% identity) copies of two HERV15 proviruses in the human Y chromosome has been shown to cause chromosomal deletion and loss in the azoospermia factor a (AZFa), leading to spermatogenic failure and infertility [57]. Moreover, oncogenic gene fusions involving the ETS (E26 transformation-specific) gene family are created in human prostate cancer by recurrent chromosomal translocation and one of the 5′ fusion partners for such translocations was identified as a HERV-K provirus [58]. A chromosomal rearrangement involving a HERV provirus has also been described in the inactivation of the mismatch repair endonuclease *PMS2* gene, loss of which predisposes to mismatch repair cancer syndrome and colorectal cancer [59].

The real potential of HERVs to cause chromosomal rearrangements by non-allelic recombination notwithstanding, the relative risk of such an event involving HERVs should be weighed against similar events involving non-LTR retrotransposons. There are 4.6 non-LTR elements for every LTR element in the human genome [9] and their overabundance makes them a better substrate for non-allelic recombination than LTR elements [28,33,47]. Moreover, the considerably greater sequence diversity among LTR elements, in comparison with non-LTR elements, further reduces the theoretical probability of non-allelic recombination between homologous HERVs.

### (c) Cellular gene expression

Even without producing their own proteins, HERVs can exert a powerful influence on host gene expression in several ways [60]. The most common is through the promoter activity of their LTRs. Indeed, HERV LTRs are targets for numerous DNA binding proteins, which have a demonstrable role in oncogenesis [17,61]. These include p53, with a third of all its genomic binding sites in HCT116 human colorectal cancer cells mapping to HERV LTRs, particularly enriched in the LTR10 and MER61 subgroups of HERVs [62].

LTRs may operate as primary or alternative promoters in nearly a third of human transcripts [60]. LTRs can initiate expression of chimeric transcripts, encompassing both HERV and cellular gene sequences, with important consequences for gene expression and function. Where the downstream cellular gene is an oncogene, *in cis* activation by the LTR will certainly contribute to oncogenesis [61]. A well-documented examples is expression of *colony-stimulating factor 1 receptor* (*CSF1R*), which in healthy haematopoietic cells is driven by its own promoter, whereas in Hodgkin's lymphoma cells it is driven by an upstream LTR, promoting survival of cancer B cells by excess CSF1R expression [63]. Alternative transcription, initiated by derepressed intronic LTR elements, has been shown to produce truncated oncogenic forms of tyrosine kinases, such as the *anaplastic lymphoma kinase* (*ALK*) in a subset of melanomas and other cancer types [64], and the *erb-b2 receptor tyrosine kinase 4* (*ERBB4*) in ALK-negative anaplastic large-cell lymphoma (ALCL) [65]. Alternative splicing of a chimeric transcript may also diminish or abolish protein function, as in the case of the human *CD5* gene, where transcription initiation from an upstream LTR results in a truncated non-functional CD5 protein in B cells [66].

There are several other examples where neighbouring LTRs alter cellular gene expression (table 1), including the *GSDML* (*gasdermin-like protein*) gene in healthy and cancer cells [67]; the *DNAJC15* (*DnaJ heat shock protein family (Hsp40) member C15*) gene in cancer cell lines [68]; the *NAIP* (*NLR family apoptosis inhibitory protein*) gene in healthy testis [69]; the *FABP7* (*fatty acid binding protein 7*) gene in diffuse large B-cell lymphoma (DLBCL) cells [70]; the *IRF5* (*interferon regulatory factor 5*) gene in Hodgkin's lymphoma cells [71]; and the *CADM2* (*cell adhesion molecule 2*) and *SEMA3A* (*semaphorin 3A*) genes in erythroleukaemia and human embryonic stem cells [72,73]. In some of these cases, LTR-driven transcription appears central to the oncogenic process [61], whereas in others it may represent a consequence of the extensive loss of epigenetic control that characterizes cellular transformation [77]. Indeed, such global epigenetic derepression of retroelements in cancer is expected to induce many more examples of chimeric transcript formation.

**Table 1.** LTR-driven expression or misexpression.

LTR element	cellular gene	effect	cancer type	study
MaLR-THE1B	*CSF1R*	overexpression	Hodgkin's lymphoma	[63]
LTR16B2	*ALK*	truncation	melanoma	[64]
MaRL- MLT1H2	*ERBB4*	truncation	ALK-negative anaplastic large-cell lymphoma	[65]
HERV-E	*CD5*	alternative splicing with loss of function	healthy B cells and chronic lymphocytic leukaemia cells	[66]
HERV-H	*GSDML*	overexpression	healthy tissues and cancer cell lines	[67]
HERV-L33 HERV-H	*DNAJC15*	ectopic expression	cancer cell lines	[68]
HERV-P	*NAIP*	not tested	healthy tissue (testis-specific)	[69]
HERV-E	*FABP7*	overexpression	DLBCL (diffuse large B-cell lymphoma)	[70]
LOR1a	*IRF5*	overexpression	Hodgkin's lymphoma	[71]
ERV9	*CADM2*	alternative splicing	stem cells and erythroleukaemia cell line	[72,73]
ERV9	*SEMA3A*	alternative splicing	stem cells and erythroleukaemia cell line	[72,73]
ERV9	*TNFRSF10B*	restored expression	healthy tissue (testis-specific)	[74]
ERV9	*TP63*	restored expression	healthy tissue (testis-specific)	[75]
MER41	*AIM2*	interferon inducibility	healthy cells	[76]

It should be noted that LTR-driven transcription of cellular genes can also have an anti-oncogenic effect. For example, expression of several cellular genes is regulated by upstream LTRs belonging to the ERV9 group of HERVs, and these include the *TP63* and *TNFRSF10B* genes, encoding the p63 homologue of the tumour suppressor p53 and death receptor 5 (DR5), respectively [74,75]. Importantly, the *TP63* (*tumour protein p63*) and *TNFRSF10B* (*TNF receptor superfamily member 10b*) genes are suppressed in primary human testicular cancer cells or cell lines, likely permitting tumour growth. However, treatment with inhibitors of histone deacetylases has been shown to reverse the epigenetic repression of the ERV9 LTR promoters, leading to induction of *TP63* and *TNFRSF10B* transcription and ultimately promoting apoptosis of testicular cancer cells [74,75].

In addition to gene deregulation as a result of epigenetic derepression, LTR promoter activity appears to have been co-opted in the interferon response network induced under physiological conditions, as exemplified in the *AIM2* (*absent in melanoma 2*) gene [76]. An LTR of the MER41 group of elements upstream of the human *AIM2* gene confers interferon inducibility, necessary for the innate immune response to cytosolic DNA [76].

The listed examples involve LTR elements that are fixed in the human germline and their role in oncogenesis should, therefore, be considered contributory, rather than causal [78]. However, the recent discovery of polymorphic non-reference HERV-K(HML-2) proviruses offers a means by which HERV activity can affect some individuals, but not others, depending on genotype [26]. Some of the newly described HERV-K(HML-2) insertions are located near cellular genes, with the potential to affect gene expression or function [26].

LTR promoter activity is not the only proposed mechanism by which HERVs can drive oncogene expression. In human melanoma cells, RNA transcribed from an MER11C element (HERV-K11 group) or from an L1PA16 element (LINE1 group) binds to and inhibits the pre-mRNA splicing factor PSF [79], whose function is to suppress expression of several oncogenes. Thus, by repressing the PSF tumour suppressor, MER11C has been shown to exert oncogenic activity [79].

Taken together, the available data suggest that epigenetic derepression of LTR elements in the context of cancer can uncover their potential to disrupt regulatory gene expression networks and promote, if not initiate, the oncogenic process.

### (d) Tumour-promoting human endogenous retrovirus proteins

Although no HERV-encoded protein can be described as acutely oncogenic, some have been suggested to aid cellular transformation or facilitate immune evasion. However, the molecular mechanisms by which HERV proteins may contribute to cancer development, remain obscure.

Embedded in the transmembrane (TM) subunit of retroviral envelope glycoproteins is the putative immunosuppressive domain, which has been proposed to promote tumour growth by suppression of anti-tumour immunity [80]. This activity has been described for syncytin-2, one of the two HERV envelope glycoproteins exapted for cellular fusion during placentation [80], as well as for the envelope glycoprotein of HERV-H [81], HERV-E [82] and HERV-K [83]. In a recent study of colon cancer, expression of HERV-H was found elevated and was required for the production of the chemokine CCL19, which, in turn, recruits and expands immunoregulatory cells [84]. Importantly, a peptide corresponding to the immunosuppressive domain of HERV-H envelope glycoprotein was sufficient to induce CCL19 production by tumour cells, as well as epithelial-to-mesenchymal transition, a process known to facilitate metastasis [84]. In addition to mediating immunosuppression, cell fusion events driven by the elevated expression of syncytins in human cancer are also suspected to play a role in transformation or metastasis [85,86].

Multiple splicing of the *env* mRNA of certain HERV-K(HML-2) proviruses produces a transcript, *rec*, which encodes the accessory protein Rec [87]. This protein is thought to be important for mediating the nuclear export of the unspliced viral RNA transcript of HERV-K(HML-2) proviruses and is variably expressed in transformed, as well as healthy cells [12,88]. Rec interacts with the promyelocytic leukaemia zinc finger protein and its overexpression supports cellular transformation [89,90].

As with their effects on cellular gene expression, potentially oncogenic HERV proteins are produced by non-polymorphic proviruses and can be expressed in non-transformed cells, which may restrict their effects to cancer promotion, rather than initiation [78].

In summary, the case for the involvement of HERVs was largely circumstantial and based on the prior examples of retroviral carcinogenesis caused by new proviral integrations. This does not seem to be the case for HERVs. Instead, any role in causation of human cancers seems likely to be subtler, possibly mediated by epigenetic changes.

## 4. The case for the defence: potential anti-oncogenic properties of human endogenous retroviruses

It is now becoming clear that oncogenesis is not simply a cell-intrinsic process. Interaction of transformed cells with neighbouring non-transformed cells, especially of the immune system, plays a decisive role in the tumour growth or metastasis and HERV products have the potential to affect such interactions.

### (a) Human endogenous retrovirus products in tumour antigenicity

The recent successes of cancer immunotherapy [91] reinforce a long-postulated role for adaptive immunity as a formidable barrier to tumour development. Initiation of adaptive immune responses to transformed cells relies on the recognition of tumour-restricted antigens. Two main sources of tumour-restricted antigen are typically targeted by the immune response, each with unique advantages and disadvantages [92]. The first comprises neo-antigens, created by non-synonymous mutations in protein-coding regions during the process of transformation, which are therefore highly tumour-specific and against which no immunological tolerance has been established [92]. The second comprises non-mutated self-proteins, often collectively referred to as cancer-testis antigens (CTAs), typically not expressed in healthy cells with the exception of the germplasm, but highly upregulated in many, if not most cancer types [93].

Owing to global epigenetic repression of endogenous retroelements in the genome, high levels of HERV expression are prevented in the majority of non-transformed cells, thus resulting in only partial immunological tolerance [94]. However, in the altered epigenetic environment of cancer cells, HERVs may be released from epigenetic repression and expression of several distinct HERV group members has been found upregulated characteristically in specific cancer types, such as melanomas expressing HERV-K(HML-6), renal cell carcinomas expressing HERV-E or seminomas expressing HERV-K(HML-2) [10–13].

Importantly, such cancer-specific HERV protein expression can ultimately lead to induction of T-cell and B-cell responses against HERV-encoded antigens [13,94–97]. There are several well-documented examples of HERV-encoded antigens targeted by T cells in a variety of cancers (table 2), although their role in preventing tumour growth has not always been established. CD8^+^ T cells target an HERV-K-encoded epitope and effectively lyse melanoma cells *in vitro* [98]. Expression of the HERV-K transcript encoding this epitope (termed HERV-K-MEL was found to be largely restricted to cutaneous and ocular melanoma, with some expression also in testis and naevi [98]. CD8^+^ T-cell reactivity to a defined HERV-K-derived epitope was also detected in patients with a past history of seminoma, and also in a minority of healthy individuals [99]. Tumour regression following haematopoietic stem-cell transplantation in renal cell carcinoma patients was associated with CD8^+^ T-cell responses to a HERV-encoded epitope [100]. Notably, this epitope was produced by a single HERV-E provirus of chromosome 6, whose expression was restricted to the clear cell variant of renal cell carcinoma and was absent from healthy cells or other types of tumour cells [97]. Expression of an X-linked HERV-H provirus, typically restricted to a subset of gastrointestinal cancers, served as the target for CD8^+^ T cells, which could recognize and lyse colorectal carcinoma cell lines, based on the endogenous expression of this particular HERV-H provirus [101].

**Table 2.** HERV-derived T-cell epitopes targeted in cancer.

LTR element	immune effectors	cancer type	study
HERV-K-MEL	CD8^+^ T cells	melanoma	[98]
HERV-K	CD8^+^ T cells	seminoma	[99]
HERV-E	CD8^+^ T cells	renal cell carcinoma	[100]
HERV-H	CD8^+^ T cells	colorectal carcinoma	[101]

The list of T-cell-targeted cancer-restricted epitopes encoded by HERVs will inevitably grow with our increasing understanding of cancer-specific immune responses. However, the contribution or ability of HERV-specific T-cell responses to prevent tumour occurrence, growth or metastasis is more difficult to quantify. Moreover, whether immune reactivity directed against HERV-encoded antigens represents a rare consequence of HERV dysregulation in cancer, or whether it is part of an evolutionarily selected tumour immunosurveillance network, is currently unclear. It has been argued that the usefulness of T-cell responses to HERV-encoded cancer-restricted epitopes might be limited by partial immunological tolerance, reducing the avidity of HERV-specific T cells [92]. To what extent HERV-mediated central tolerance reduces the avidity or precursor frequency, and therefore general usefulness of HERV-specific T cells that could be protective against cancer has not yet been established. Nevertheless, the specific examples listed above do suggest that HERV-specific T cells with potent anti-cancer activity can be found in the natural T-cell repertoire. Moreover, T cells can be engineered to carry higher avidity HERV-specific receptors and could be deployed in a therapeutic setting. Indeed, T cells carrying a chimeric antigen receptor derived from a monoclonal antibody against HERV-K envelope glycoprotein showed significant anti-tumour activity in human melanoma xenograft mouse model [102].

Adaptive immunity to cancer-expressed HERV antigens is not restricted to cellular responses. Although also occasionally detected in seemingly healthy individuals, HERV-specific antibodies are frequently elevated in cancer. In fact, it was the induction of HERV-K-reactive antibodies in the sera of patients with germ cell tumours that facilitated the initial discovery of HERV-K proteins and virions produced in teratocarcinoma cells lines [103]. The specificity of HERV-reactive antibodies that have been detected in cancer patients seems to be highly restricted to proteins expressed by members of the HERV-K group (table 3), but this might be a consequence of greater availability of HERV-K reagents, relatively to those for other HERV groups. In addition, being the most recent group to invade the human germline, a higher proportion of HERV-K proviruses contain functional open reading frames than other groups. Indeed, six of a total of 19 codogenic *env* genes in the human genome belong to the HERV-K group [111]. HERV-K-reactive antibodies have been detected in the sera of patients with germ cell cancers [104,105,112], prostate cancer [106], melanoma [107–109] or breast cancer [110]. Monoclonal antibodies to HERV-K envelope glycoprotein have shown therapeutic potential in a human breast cancer xenograft mouse model [113], implying a similar anti-cancer effect of such antibodies in cancer patients. However, serum titres of HERV-K-reactive antibodies correlate positively with disease activity in humans, both in patients with germ cell tumours [112] and in those with melanoma [108]. These findings suggest that humoral responses against HERV-K antigens might either simply reflect or even contribute to disease severity, a link that will need to be further investigated.

**Table 3.** HERV-derived B-cell antigens targeted in cancer.

LTR element	target protein	cancer type	study
HERV-K	Gag	seminoma	[104]
HERV-K	Env TM subunit	germ cell cancer	[105]
HERV-K	Gag	prostate cancer	[106]
HERV-K	Env Gag	melanoma	[107–109]
HERV-K	Env	breast cancer	[110]

### (b) Human endogenous retrovirus products in tumour immunogenicity

Although necessary, the availability of cancer-specific antigens alone is not sufficient to elicit an anti-cancer immune response. Such antigens need to be presented in an immunogenic, rather than a tolerogenic context. Tumour cells may employ diverse mechanisms of immune evasion or suppression, but the requirements for induction of a robust and protective anti-tumour response are still incompletely understood. Recent evidence suggests that the intrinsically viral nature of HERVs may activate an innate antiviral state that enhances tumour immunogenicity.

When correlates of anti-tumour immunity were investigated in a comprehensive study of thousands of high-dimensional cancer datasets [114], a potential link with HERV activity was revealed. Indeed, by comparing tumour samples with the respective healthy tissue, this study confirmed the largely tumour-restricted expression of numerous HERVs [114]. Importantly, HERV transcriptional activation in multiple cancer types positively correlated with intratumoural immune cytolytic activity [114]. The mechanisms underlying this association are not yet fully understood, but they may involve the synthesis of HERV-encoded epitopes that trigger adaptive immunity, as well as activation of innate immune sensors by HERV replication intermediates [94].

Support for a role of HERVs in enhancing tumour immunogenicity through activation of innate immunity is also provided by studies of the anti-cancer drug 5-azacitidine, a DNA demethylating agent used primarily in the treatment of myelodysplastic syndrome and other myeloid cancers. Treatment with 5-azacitidine has been suggested to enhance tumour antigenicity by upregulating expression of CTAs, which become targets for adaptive immunity [115–117]. However, 5-azacitidine may also enhance tumour immunogenicity by transcriptional activation of HERVs, which subsequently trigger innate immunity [118,119]. The DNA hypomethylation state induced by 5-azacitidine treatment of colorectal cancer cells [119] and of epithelial ovarian cancer cells [118] has recently been shown to activate transcription of HERVs, whose replication intermediates trigger a cancer cell-intrinsic antiviral state, characterized by upregulation of interferon response genes. The molecular characteristics of HERV replication intermediates that might trigger innate immune sensors are still poorly characterized [94,120], but they are postulated to involve sensing of double-stranded RNA by the TLR3 or the MDA5-MAVS signalling cascades [118,119].

Although such an antiviral can reduce tumour cell proliferation in a cell-intrinsic manner, these studies also demonstrate induction of a stronger anti-tumour immune response as a result of 5-azacitidine treatment [118,119]. Interestingly, 5-azacitidine treatment also induced an interferon response and set an antiviral state in murine B16 melanoma cells *in vitro* [119] and directly enhanced immune rejection of murine B16 melanoma cells in combination with immune checkpoint blockade *in vivo* [118].

Notably, B16 melanoma cells are repeatedly re-infected with and produce high levels of a fully infectious murine leukaemia virus, MelARV, which arose through recombination of defective endogenous precursors [36]. MelARV expression in B16 melanoma cells has been found necessary to prevent their immune rejection and promote their growth *in vivo* [121]. Thus, if 5-azacitidine induces an antiviral state in B16 cells through transcriptional activation of ERVs, then the innate immune stimulating effect of these ERVs must considerably outperform that of the fully infectious MelARV, already present in the cells. Although currently incompletely understood, the precise mechanisms by which HERVs potentiate tumour cell immunogenicity may hold the key to understanding, as well as manipulating, anti-cancer immunity.

In summary, it is becoming clear that HERV-encoded proteins represent potential targets for immune-mediated mechanisms of tumour control. Stimulating such responses has the potential for significant improvements in this area.

### (c) Human endogenous retrovirus proteins with potential anti-tumour activity

In addition to providing targets for immune recognition, HERV-encoded proteins may exert biological activities that could potentially inhibit tumour development. Although their primary function is to mediate entry into a target cell by binding to their respective receptors and mediating membrane fusion, envelope glycoproteins can also interact with their cellular receptors in the producer cell. Such interaction can lead to sequestration of the cellular receptors away from the plasma membrane of the infected cell, which in turn provides resistance to superinfection with retroviruses using the same cellular receptor [122]. The *env* open reading frame of single-copy HERV-T provirus in the human genome appears to have been under positive selection for rendering cells resistant to infection with the now extinct exogenous precursor of HERV-T proviruses [123]. The cellular receptor for the HERV-T envelope glycoprotein has recently been identified as the monocarboxylate transporter-1 (MCT-1), a nutrient transporter that might be required for optimum growth of transformed cells [123]. This raises the intriguing possibility that depletion of MCT-1 from the plasma membrane, as a result of HERV-T envelope glycoprotein expression, restrains tumour growth by limiting nutrient transport, a suggestion that warrants further investigation.

## 5. Concluding remarks

As a whole, endogenous retroelements have been intricately linked to the development of cancer. The incriminating evidence stems from either the potentially oncogenic behaviour of infectious retroviruses or the genomic mobility retained only by certain groups of endogenous retroelements. Thus, it would appear that HERVs are rather hastily found guilty of a causative role in cancer either by their conspicuous expression at the scene of the crime or by association with other endogenous retroelements, such as their infectious predecessors in ancestral species and other non-LTR retroelements still active in humans. However, the extraordinary depth at which cancer genomes are currently analysed suggests that HERVs have entirely lost their ability to cause cancer by mechanisms involving novel integration leading to insertional mutagenesis.

HERVs may still contribute to the pathological processes leading to cancer by other mechanisms. However, such contribution should be weighed against a potential role for HERVs in preventing tumour development. Although still only poorly understood or appreciated, the latter processes involve enhancement of tumour antigenicity and immunogenicity, afforded by elevated expression of HERV nucleic acid and protein products in cancer cells. Such as role for HERVs is akin to an in-built ‘warning system’, alerting the cell, as well as the immune system, to any genetic damage or epigenetic dysregulation.

## Data accessibility

This article has no additional data.
